# Integrated assessment of brackish water induced deleterious impacts: Analysis of hemato-biochemistry, erythrocytic toxicity, histopathology and oxidative stress biomarkers of mrigal carp

**DOI:** 10.1371/journal.pone.0338381

**Published:** 2026-01-23

**Authors:** Ghulam Ali Raza, Riaz Hussain, Abdul Ghaffar, Mudassar Mohiuddin, Roshan Riaz, Shah Nawaz, Mükremin Ölmez, Dalia Fouad, Promy Virk

**Affiliations:** 1 Department of Zoology, The Islamia University of Bahawalpur, Bahawalpur, Pakistan; 2 Department of Pathology, Faculty of Veterinary and Animal Sciences, The Islamia University of Bahawalpur, Bahawalpur, Pakistan; 3 Department of Microbiology, Faculty of Veterinary and Animal Sciences, The Islamia University of Bahawalpur, Bahawalpur, Pakistan; 4 Department of Animal Nutrition and Nutritional Disease, Faculty of Veterinary Medicine, Kafkas University, Kars, Türkiye; 5 Department of Comparative Biomedical Sciences, School of Veterinary Medicine, Faculty of Health and Medical Sciences, University of Surrey, United Kingdom; 6 Department of Zoology, College of Science, King Saud University, Riyadh, Saudi Arabia; CIFRI: Central Inland Fisheries Research Institute, INDIA

## Abstract

Salinity is recognized as one of the most detrimental abiotic stressors, inducing a range of cellular and biochemical disruptions in both marine and freshwater organisms worldwide. In this study, 84 healthy specimens of mrigal carp (*Cirrhinus mrigala*) were randomly selected and assigned to four experimental groups (T0, T1, T2, and T3). Each group was maintained under standardized laboratory conditions and exposed to different salinity levels (0, 3, 5, and 7 ppt) in brackish water to evaluate the resulting toxico-physiological responses over 90 days interval. Hematological analysis revealed a significant reduction in erythrocyte count, hemoglobin concentration, hematocrit values, and lymphocyte percentage in salinity-exposed (5, and 7 ppt) fish. In contrast, a notable elevation in total white blood cell (WBC) count and neutrophil population was observed in fish exposed to salinity (5, and 7 ppt) at days 60 and 90 of study. Biochemical assessment of serum parameters demonstrated a marked decline in albumin triglycerides and glucose levels, whereas concentrations of lactate dehydrogenase (LDH), cholesterol, alanine aminotransferase (ALT), aspartate aminotransferase (AST), alkaline phosphatase (ALP), creatinine, and urea increased significantly in fish at days 60 and 90 of trial subjected to salinity (5, and 7 ppt). A significant elevation in oxidative stress biomarkers was observed in fish exposed to brackish water (5, and 7 ppt), accompanied by a substantial decline in the activity of key antioxidant enzymes, including glutathione (GSH), catalase (CAT), and superoxide dismutase (SOD) in erythrocytes. Histopathological examination of visceral organs—such as the gills, kidneys, liver, brain, and heart—revealed pronounced tissue damage and pathological alterations, particularly in fish exposed to higher salinity levels (5and 70 ppt). Overall, the findings indicate that elevated salinity in brackish water can provoke oxidative stress, impair hematological and biochemical homeostasis, suppress antioxidant defense mechanisms in erythrocytes, and induce microscopic ailments in different visceral organs of fresh water fish in a time dependent response. In conclusion, hematological and biochemical biomarkers are useful tools and provide reliable information about immune potential, level of stress, intoxication and severity of histopathological lesions.

## 1. Introduction

Aquaculture is rapidly advancing as a sustainable means of enhancing the global supply of diverse food sources and high-quality proteins, offering significant potential in combating malnutrition worldwide [1–4]. It serves as a vital strategy for meeting the increasing global demand for food and addressing nutritional deficiencies [5–7], as well as having a lesser environmental effect than many other animal-based foodstuffs [8,9]. Aquaculture and related practices play a pivotal role in supporting the conservation and expansion of biodiversity among various aquatic species [10–12], and can provide a variety of resources to public health across the globe including dietary energy, water, recreation, and transportation [13,14]. Abrupt and unpredictable climatic variations within aquatic ecosystems are recognized as major stressors that adversely affect the optimal growth and productivity of aquaculture species [15,16]. 2022). Brackish water is characterized by salinity levels that are higher than freshwater but lower than seawater [17]. Major sources of brackish water include naturally saline aquifers, river systems, wastewater from industrial processes (such as cooling operations, hydraulic fracturing, and other anthropogenic activities), as well as return flows from agricultural irrigation [18,19]. The formation of brackish water is often facilitated by the runoff of saline minerals such as gypsum and halite from sedimentary rock formations, as well as by industrial discharges containing dissolved salts and leachates from saline soils [20–22]. Various reports estimate that salinity adversely affects nearly one-third of the world’s arable land, posing a significant threat to agricultural productivity and soil health [23,24].

Escalating salinization—evidenced by increasing concentrations of inorganic ions in aquatic environments—has emerged as a critical environmental concern [25,26]. Salinization can occur naturally (primary salinization) or as a result of human actions (secondary salinization) [27]. Mining activities, industrial effluents, the application of road salts, and agricultural irrigation practices are among the leading contributors to anthropogenic salinization of water bodies [28–30]. Elevated salt concentrations originating from various anthropogenic and natural sources can accumulate in adjacent water bodies, such as streams and rivers, exerting significant physiological stress on exposed aquatic organisms [31], particularly in semi-arid and arid regions [32]. Globally, salinization affects approximately 1,125 million hectares of land, with around 76 million hectares impacted directly by human activities [33]. Elevated salinity levels, or hyper-salinity, adversely affect the development, osmoregulatory functions, physiological state, and immune responses of freshwater species [34,35]. Among the various assessment tools, blood biomarkers are considered highly reliable and sensitive indicators for detecting and monitoring environmental contaminants, including salts, as they reflect the organism’s internal response following direct absorption of pollutants into the bloodstream [36,37]. Blood biomarkers offer critical insights into the physiological and biochemical status of both target and non-target aquatic organisms, including fish, in response to environmental fluctuations and the presence of waterborne contaminants [12,38–41]. Salinity is widely recognized as one of the most detrimental abiotic stressors affecting aquatic animals globally, leading to a range of cellular and biochemical alterations [42]. Previous studies have reported that abrupt salinity changes—especially in estuarine and coastal regions caused by shifts in weather patterns and rainfall—can markedly affect fish survival and productivity [43]. The assessment of serum biomarkers is considered a critical approach for evaluating the physiological status of fish, determining the health of visceral organs, and monitoring oxidative stress responses in both aquatic and terrestrial species [44,45]. Elevated oxidative stress leads to a reduction in antioxidant enzyme activity and tissue protein content, resulting in damage to cellular lipids, DNA, and proteins. Furthermore, variations in the physicochemical properties of water have been shown to profoundly impact the growth, anatomy, and morphology of fish [46]. Histological and morphological changes are particularly evident in the gills and digestive system, which are in direct contact with the aquatic environment and are highly sensitive to ecological disturbances [47]. *C. mrigala* is a member of Cyprinidae family of fresh water fish. It is frequently cultured in different parts of world including Nepal, Bangladesh, Pakistan, and India due to high quality meat and taste [48]. Therefore, the present study was conducted to evaluate the hematological, serological, histopathological, and oxidative stress responses in the erythrocytes of mrigal carp (*Cirrhinus mrigala*) reared under varying salinity conditions in brackish water.

## 2. Methodology

### 2.1. Experimental species and management

The present experimental trial was conducted in the laboratories of the Department of Pathology and Zoology at the Islamia University of Bahawalpur, Punjab, Pakistan. All the protocols and experimentations were approved by the committee on ethics and animal welfare ensuring the ethical treatments and welfare of laboratory animals, Department of Zoology and advanced studies and research board (925/AS&RB 10/10/ 2022) of the Islamia University of Bahawalpur. A total of 84 healthy mrigal carp (*Cirrhinus mrigala*), with uniform body weights ranging from 160 to 180 grams, were procured from a commercial fish breeding center located in Bahawalpur District. Following collection, the fish were transported to the laboratory in oxygenated plastic bags to minimize handling stress. Upon arrival, the fish were acclimatized for 16 days in glass aquaria (75 cm × 45 cm × 45 cm) under standardized laboratory conditions. Fish were kept at standard housing conditions under controlled temperature (24 ± 1ºC), pH (7.5) and continuous aeration at dissolved oxygen level of 6.16 ppm using aquarium air pump (SOBO SB-548A). The fish were randomly assigned to four experimental groups (T0, T1, T2, and T3), with each group comprising 21 individuals. All fish were fed a commercial diet containing 25% crude protein, administered twice daily (morning and evening). Water quality was maintained by performing weekly water changes, and residual feed along with fecal matter was removed daily to ensure hygienic conditions. Key physicochemical parameters of the water were monitored and recorded on days 30, 60, and 90 of the experimental periods.

### 2.2. Experimental treatments

Brackish water having 7.2 ppt was taken from Derawar fort (Cholistan), Punjab, Pakistan, and diluted to 3.00 ppt, 5.00 ppt, and 7.00 ppt by adding fresh water. After acclimatization, the fish in different groups (T0, T1, T2 and T3) were exposed to brackish water in glass aquaria (75 cm × 45 cm × 45 cm) having various levels of salinity T0 (0.0 ppt), T1 (3.00 ppt), T2(5.00 ppt) and T3 (7.00 ppt) for three months. Fish reared in group T0 were placed in fresh water (0.00 ppt) as untreated control. The body mass of fish reared in each normal and exposed groups was recorded at days 30, 60 and 90 of trial. Therefore, salinity concentrations used in this experimental study were used on the basis of earlier published reports [49–51]. All the fish were critically observed for any physical/behavioral disorders on regular basis throughout the trial.

### 2.3. Hematological studies

To minimize the stress prior to blood collection, all the fish were fasted for 10-12h and were handled gently in a calm and shaded area. Prior to blood collection, the experimental mrigal carp were anesthetized to alleviate any kind of stress using clove oil (4–5 mg/L). The fish were monitored continuously until anesthesia was reached (loss of reflexes and absence of movement). After that, approximately 2 mL blood was collected at days 30, 60, and 90 from the caudal vein of each fish with the help of 26-gauge sterile needle. The collected blood was immediately transferred into test tubes both with and without anticoagulant EDTA (1.2 mg/ml) depending on the intended analysis. Hematological parameters were assessed following the protocols described [52]. The evaluated biomarkers included erythrocyte count (×10^9^/mm^3^), hematocrit (%), hemoglobin concentration (g/dL), total leukocyte count (×10⁶/mm^3^), and differential leukocyte counts—specifically neutrophils (%), monocytes (%), and lymphocytes (%). These assessments were performed in accordance with the methods outlined [53,54].

### 2.4. Serum chemistry

Serum was separated from blood samples collected from fish in each experimental group on days 30, 60, and 90 by placing the samples on ice and allowing coagulation, followed by centrifugation. A range of serum biochemical parameters—including aspartate aminotransferase (AST), alanine aminotransferase (ALT), alkaline phosphatase (ALP), urea, lactate dehydrogenase (LDH), glucose, creatinine, triglycerides, albumin, and cholesterol—were quantified using commercially available diagnostic kits (Randox Laboratories Pvt. Ltd.). Measurements were performed using a fully automated chemistry analyzer (Randox Laboratories Pvt. Ltd.) at each designated sampling interval, following established protocols [54,55].

### 2.5. Histopathological examination

After blood collection, the fish were then placed in another tank containing the experimental water and were euthanized using clove oil at higher concentrations to alleviate the stress. The fish were monitored continuously until euthanasia was reached and death was confirmed by loss of reflexes. loss of consciousness and absence of movement. Finally, necropsy of each fish was carried out immediately after death for collection of visceral organs. Visceral organs (liver, kidneys, brain, heart, and gills) were obtained from each experimental fish at days 30, 60 and 90 of trial for histopathological analysis. Prior to dissection, fish were anesthetized using clove oil at a concentration of 4.5 mg/L to minimize stress and ensure humane handling. Immediately after dissection, the excised tissues were rinsed in physiological saline and fixed in 10% neutral buffered formalin for preservation. Following fixation, tissues were processed through a graded ethanol series for dehydration, embedded in paraffin, and sectioned on microtome. The tissue sections were stained with hematoxylin and eosin (H&E) following standard histological protocols [54,55]. Microscopic evaluation was carried out under light microscope (Nikon Eclipse 80i, Nikon Corporation, Tokyo, Japan).

### 2.6. Status of antioxidant enzymes and oxidative stress bio markers in erythrocytes

The evaluation of thiobarbituric acid reactive substances (TBARS) was done as described by Uchiyama and Mihara [56], with slight modifications. Approximately, 2 ml of blood was collected from each fish in test tube containing anticoagulant (ethylenediaminetetraacetic acid) using sterile hypodermic needle for the estimation of different oxidative and antioxidative stress biomarkers in erythrocytes. All the collected blood samples were separately centrifuged at 5000 rpm for 10 min and plasma and buffy coat containing leukocytes was removed. After that, the remaining erythrocytes were washed using chilled normal saline and were stored at −20°C for further analysis. Finally, 10% hemolysate/precipitate of erythrocytes was prepared by adding cold deionized water in pellet of erythrocyte with slowly stirring. In brief, 0.25 mL of erythrocyte precipitate was added to 25 μL of 10 mM butylated hydroxytoluene, 3.0 mL (1%) phosphoric acid (Sigma-Aldrich), and 1.0 mL (0.67%) thiobarbituric acid solution (Sigma-Aldrich). The reaction mixture was incubated at 90 °C for 45 minutes. Following incubation, TBARS levels were measured by noting absorbance at 535 nm using a UV-visible spectrophotometer [57].

Glutathione (GSH) level in erythrocytes was determined following the protocol established by Beutler et al. [58]. Briefly, 0.1 mL of erythrocyte precipitate was diluted in 0.9 mL of double-distilled water. Subsequently, 1.5 mL of precipitating reagent—comprising NaCl, EDTA, and m-phosphoric acid—was added to the mixture. The reaction mixture was kept at room temperature conditions for 5 minute, followed by centrifugation (4000 rpm/15 minutes/4 °C). Thereafter, 1.0 mL resulting supernatant was added to 4.0 mL phosphate buffer (0.3 M) and 0.5 mL DTNB, prepared in sodium citrate (1%). The absorbance of the reaction mixture was recorded at 412 nm using a UV-visible spectrophotometer to quantify GSH concentration.

Superoxide dismutase (SOD) activity in erythrocytes was measured as described by Marklund and Marklund [59]. Blood samples were first centrifuged (2500 rpm/15 minutes/4 °C) to separate plasma, which was discarded. The remaining erythrocytes were washed with normal saline to remove residual plasma components. The washed erythrocytes were then lysed, and the lysate was used for enzymatic analysis. The reaction mixture consisted of 0.1 mL erythrocyte lysate, 30 µL pyrogallol, and 2.9 mL Tris-HCl buffer. The absorbance was recorded at 420 nm using a UV-visible spectrophotometer to determine SOD activity. Catalase activity in the erythrocyte lysate was assessed following the protocol established by Sinha [60].

### 2.7. Statistical analysis

All data are expressed as mean ± standard error (SE). Normality of the data distribution was confirmed prior to analysis. One-way analysis of variance (ANOVA) was carried out using IBM SPSS statistical software (version 20) to assess differences among the experimental groups. Tukey’s post hoc test being a reliable, sensitive, widely acceptable and less objectionable [61] was applied for comparing means (P < 0.05). This statistical approach was used to evaluate variations in serum biochemical parameters, hematological indices, antioxidant enzyme activities, and oxidative stress markers in erythrocytes between the control and treatment groups.

## 3. Results

**Fig 1:**
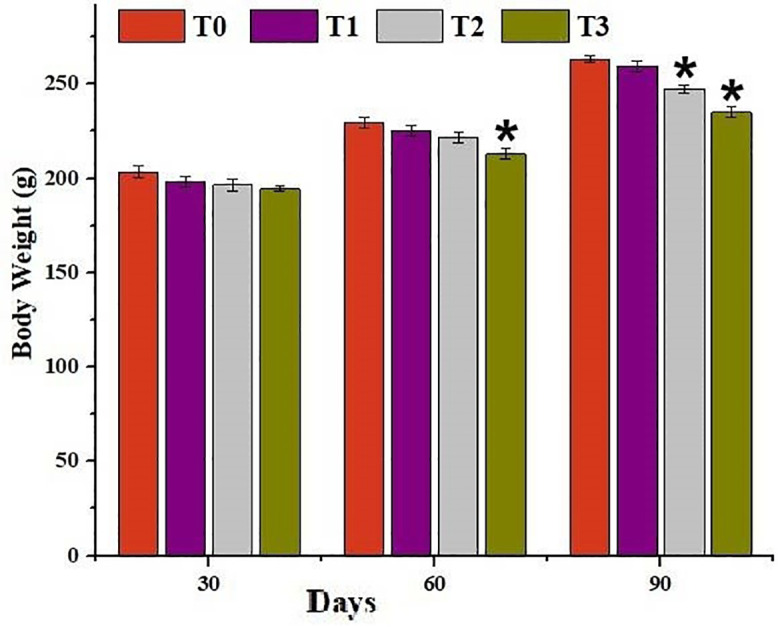
Comparison on body weight of mrigal carp (*Cirrhinus mrigala*) reared in brackish water of different salinity regimes. Bars bearing asterisk indicate significant difference (P < 0.05) from control group.

The results on growth performance of mrigal carp reared in brackish water having different salinity levels at day 30 indicated non-significant difference. The results revealed significant decrease in body mass of fish reared in group T3 at days 60 and in fish of groups T2 and T3 at days 90 as compared to control group (Fig 1).

### 3.1. Hematology

**Fig 2:**
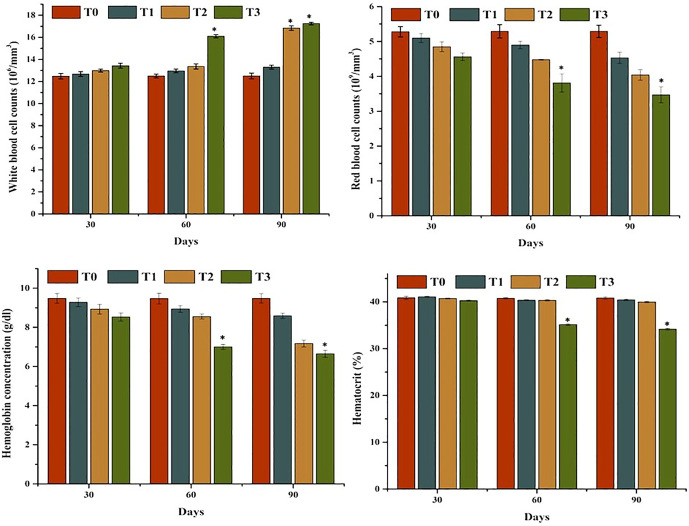
Comparison on hematological analysis of mrigal carp (*Cirrhinus mrigala*) reared in brackish water of different salinity regimes. Bars bearing asterisk indicate significant difference (P < 0.05) from control group.

**Fig 3:**
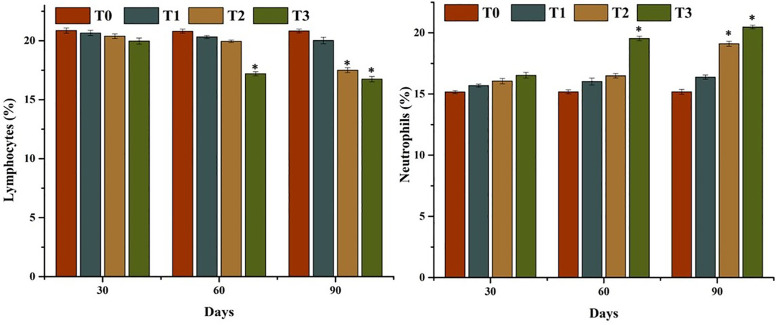
Comparison on lymphocyte and neutrophil counts of mrigal carp (*Cirrhinus mrigala*) reared in brackish water of different salinity regimes. Bars bearing asterisk indicate significant difference (P < 0.05) from control group.

The results of comparisons of hematological parameters of mrigal carp (*Cirrhinus mrigala*) reared in freshwater and varying salinity levels of brackish water showed significant variation. A significant reduction in red blood cell (RBC) count, hematocrit (%), and hemoglobin concentration (g/dL) was seen in fish exposed to higher salinity (5, and 7 ppt) levels by day 90 of the experiment (Fig 2). Lymphocyte counts declined significantly in fish from group T2 at day 90, and in group T3 at both days 60 and 90 (Fig 3). In contrast, total WBC and neutrophil counts increased significantly at days 60 and 90 in fish subjected to higher salinity (groups T2 and T3), indicating a stress-induced immunological response to elevated salinity conditions.

### 3.2. Serum analysis

**Fig 4:**
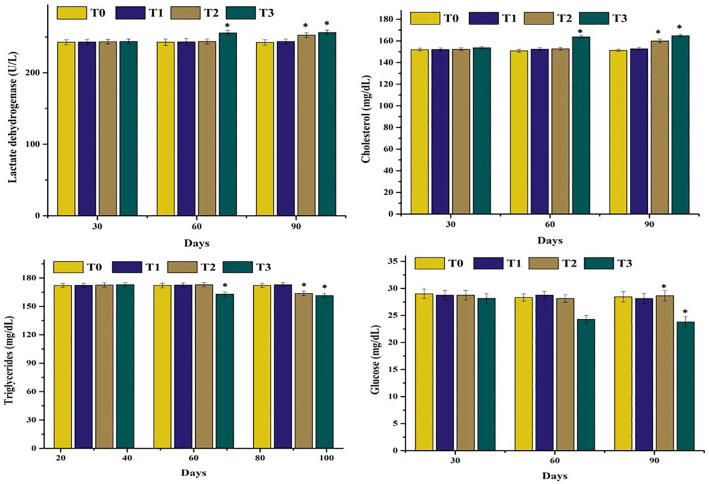
Comparison on different serum biochemistry analysis of mrigal carp (*Cirrhinus mrigala*) reared in brackish water of different salinity regimes. Bars bearing asterisk indicate significant difference (P < 0.05) from control group.

**Fig 5:**
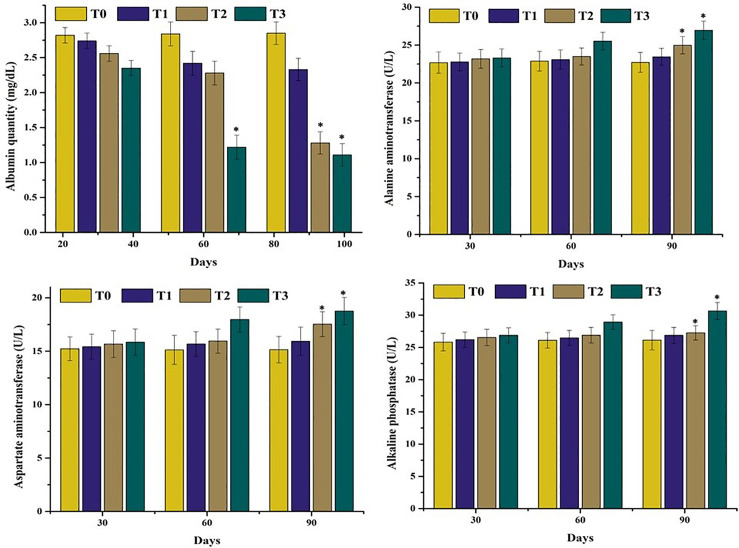
Comparison on serum albumin and different liver function tests of mrigal carp (*Cirrhinus mrigala*) reared in brackish water of different salinity regimes. Bars bearing asterisk indicate significant difference (P < 0.05) from control group.

**Fig 6:**
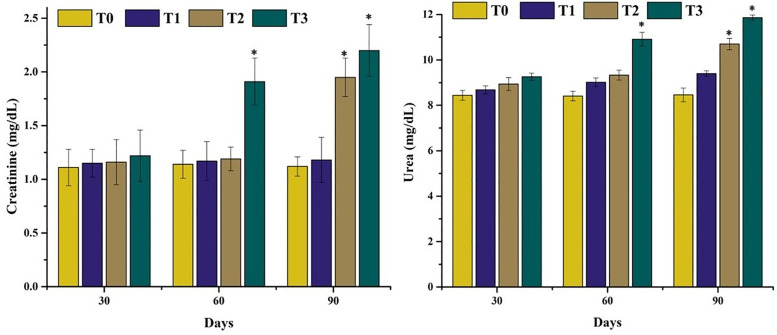
Comparison on different serum biomarkers of kidneys of mrigal carp (*Cirrhinus mrigala*) reared in brackish water of different salinity regimes. Bars bearing asterisk indicate significant difference (P < 0.05) from control group.

The results on serum biochemical parameters of mrigal carp (*Cirrhinus mrigala*) reared under varying salinity levels in brackish water indicated significant increase or decrease values as compared to normal fish. A significant increase (p < 0.05) in lactate dehydrogenase (LDH), and cholesterol while significant decrease in triglyceride and glucose levels was observed at day 90 in fish (Fig 3) exposed to 5.00 ppt salinity (T2), while these parameters were significantly increased/decreased at both days 60 and 90 in fish maintained at 7.00 ppt salinity (T3), compared to the control group (Fig 4). A marked reduction in serum albumin concentration was recorded at day 90 in group T2 and at both days 60 and 90 in group T3. Additionally, levels ALT, AST, and ALP were significantly elevated in group T3 at both days 60 and 90, indicating hepatic stress (Fig 5). Kidney function biomarkers—creatinine and urea—were also significantly increased at day 90 in group T2 and at both days 60 and 90 in group T3, suggesting renal impairment under high salinity exposure (Fig 6).

### 3.3. Status of oxidative stress parameters and antioxidant enzymes in erythrocytes of Mrigal carp

**Fig 7:**
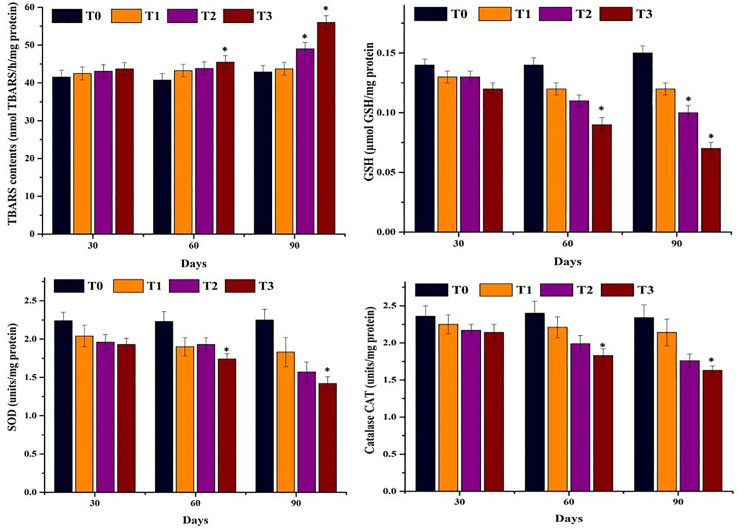
Comparison of oxidative stress parameters and antioxidant enzymes in Erythrocytes of Mrigal carp. Bars bearing asterisk indicate significant difference (P < 0.05) from control group.

Data on oxidative stress markers and antioxidant enzyme activities in erythrocytes of mrigal carp (*Cirrhinus mrigala*) exposed to different salinity levels of brackish water are presented in Fig 7. A significant increase in thiobarbituric acid reactive substances (TBARS) and glutathione (GSH) levels was observed at day 90 in fish exposed to 5.00 ppt salinity, and at both days 60 and 90 in those exposed to 7.00 ppt salinity, indicating elevated oxidative stress. Conversely, the key antioxidant enzymes – SOD and CAT—were significantly reduced at day 90 in fish reared in both 5.00 ppt and 7.00 ppt salinity treatments compared to the control group, suggesting impaired antioxidant defense under high salinity conditions.

### 3.4. Histopathology

**Fig 8:**
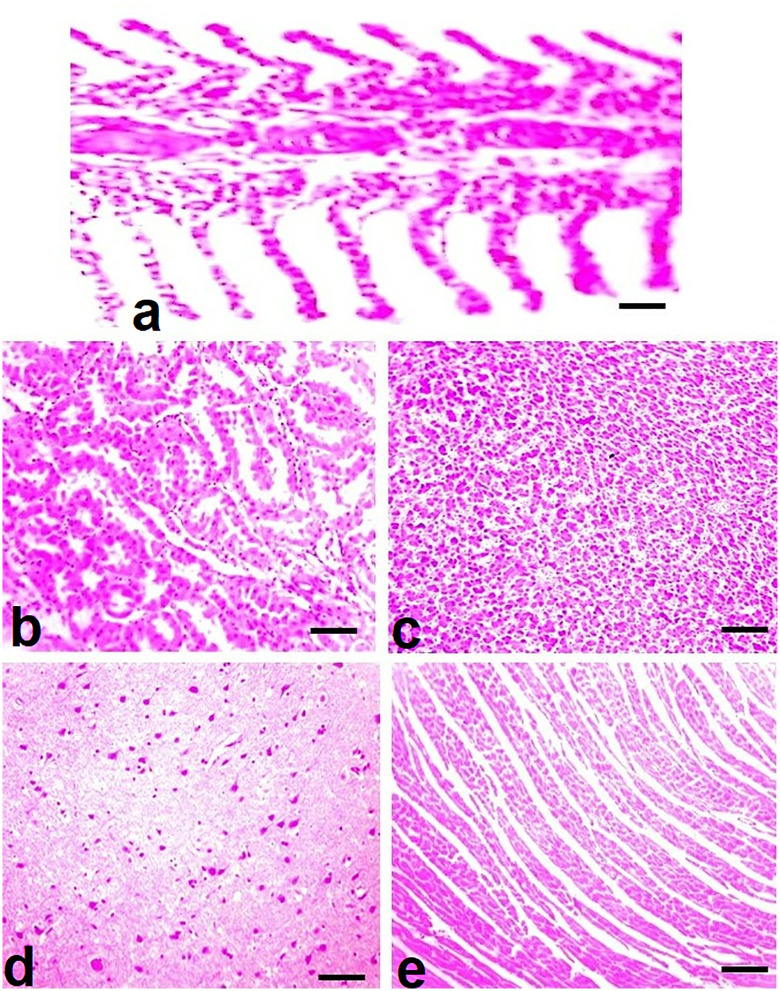
Photomicrograph of various visceral organs such as gills (a), kidneys (b), liver (c) brain (d), and heart (e) showing normal histological arrangements in control fish. H and E stain; 400X.

**Fig 9:**
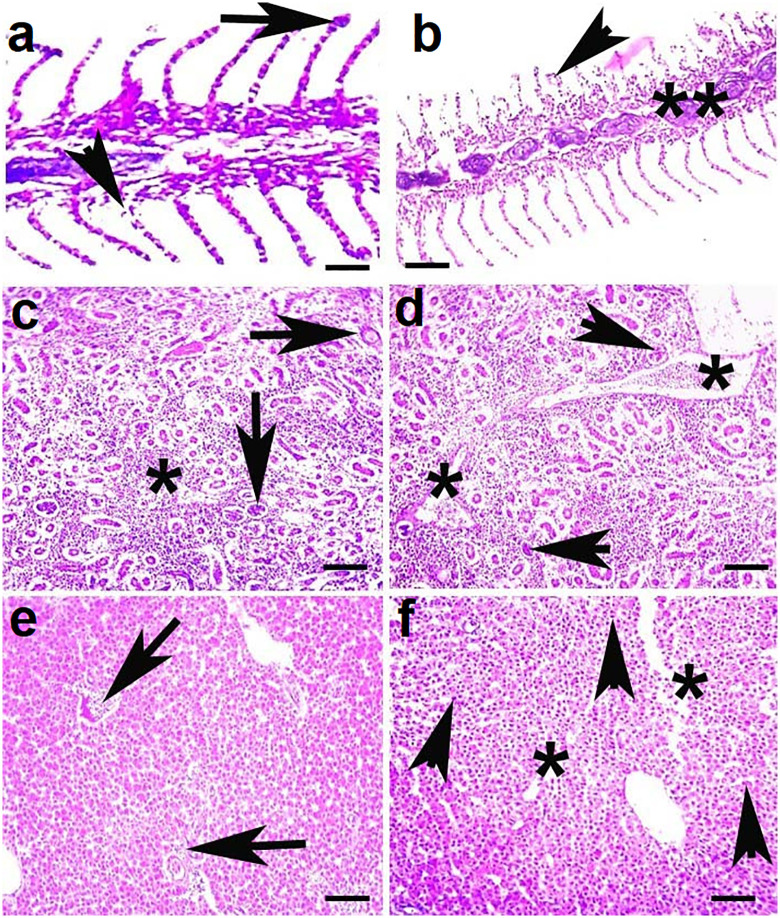
Photomicrograph of various visceral organs a) Gills exhibiting necrosis of lamellar cells (arrow), degeneration of primary and secondary lamellae and uplifting of secondary lamellae and necrosis of lamellar epithelium (arrow head) in fish reared at salinity (T2) at day 90. b) Gills exhibiting aneurysm (arrow head), necrosis of primary and secondary lamellar cells and disorganization of cartilaginous core (**) in fish reared at salinity (T3) at day 90. c) Kidneys exhibiting extensive necrosis of tubular cells, aggregates of melanomacrophages (*) and necrosis and degeneration of renal tubules (arrows) in fish reared at salinity (T2) at day 90. d) Kidneys exhibiting extensive necrosis of tubular cells, necrosis of tubules (arrow heads), inflammatory exudate (*) and degeneration of renal tubules in fish reared at salinity (T3) at day 90. e) Liver exhibiting congestion, inflammatory materials (arrows) and necrotic hepatocytes in fish reared at salinity (T2) at day 90. f) Liver exhibiting necrosis of hepatocytes (arrows), degeneration, increased sinusoidal spaces and disorganization of hepatocytes (*) in fish reared at salinity (T3) at day 90. H and E stain; 400X.

**Fig 10:**
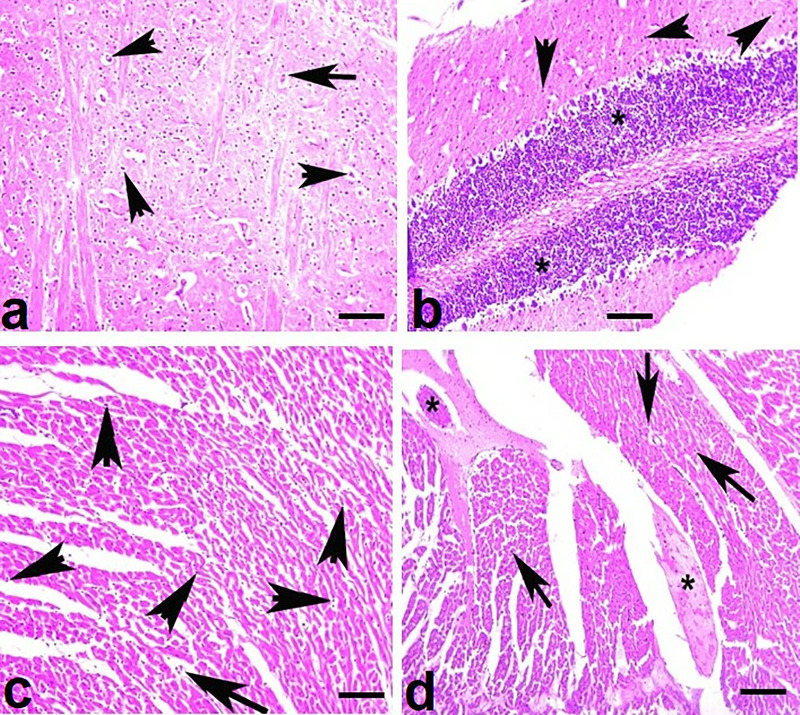
Photomicrograph of various visceral organs a) Brain showing necrosis and degeneration of neurons (arrow heads), enlarged cytoplasm and eccentric nuclei of neuron (arrow) in fish reared at salinity (T2) at day 90. **b)** Brain showing degeneration and atrophy of neurons, necrosis of neurons (arrow heads) and microgliosis (*) in fish reared at salinity (T3) at day 90. **c)** Heart showing degeneration and disorganization of cardiac muscles, necrosis of myocytes (arrow heads) and sporadic inflammatory cells (arrow) in fish reared at salinity (T2) at day 90 and **d)** Heart showing degeneration, myofibrillosis, necrosis of myocytes (arrow heads), inflammatory exudate (*) and edema in fish reared at salinity (T3) at day 90. H and E stain; 400X.

Histopathological examination of visceral organs from the untreated control group revealed normal tissue architecture across all organs analyzed (Fig 8). In contrast, fish exposed to brackish water exhibited varying degrees of microscopic lesions depending on the salinity level and duration of exposure. In the gills of fish reared at 5.00 ppt salinity, histological alterations at day 90 included lamellar cell necrosis and degeneration of both primary and secondary lamellae. More severe changes were observed in the gills of fish exposed to 7.00 ppt salinity, including lamellar cell necrosis, aneurysms, and disorganization of the cartilaginous core (Fig 9). In the kidneys, fish exposed to 5.00 ppt salinity showed extensive tubular cell necrosis and renal tubular degeneration at day 90. Similar but more pronounced lesions, such as widespread tubular necrosis, edema, and structural disintegration of renal tubules, were evident in fish reared at 7.00 ppt salinity (Fig 9). Liver sections from fish in the 5.00 ppt group displayed hepatic congestion and focal necrosis, whereas those from the 7.00 ppt group exhibited more advanced changes, including vacuolar degeneration, disorganization of hepatocyte cords, and widespread hepatocellular necrosis (Fig 9). Brain tissues of fish exposed to 5.00 ppt salinity showed necrosis of neurons and microgliosis by day 90. In the 7.00 ppt group, these alterations were more extensive, with degeneration and atrophy of neurons, pronounced neuronal necrosis, and microgliosis observed (Fig 10). Cardiac tissues from the 5.00 ppt group showed degeneration and disorganization of cardiac muscle fibers, while fish reared at 7.00 ppt salinity displayed more severe histopathological changes, including myofibrillolysis, inflammatory exudates, and edema (Fig 10).

## 4. Discussion

Fluctuations in aquatic environmental conditions, particularly due to contamination of water, can lead to a range of physiological, morphological, endocrinological, biochemical, and histopathological disturbances in fish [62,63]. Salinity-induced stress alters oxygen consumption and increases energy demands, thereby affecting the overall metabolic balance of aquatic organisms. Different fish species exhibit species-specific salinity tolerances, which influence their growth, reproductive performance, resilience to environmental challenges, and feed utilization efficiency. In this context, the present study was designed to assess the impact of brackish water with varying salinity levels on hematological and serum biochemical parameters, oxidative stress markers, antioxidant enzyme activities in erythrocytes, and histopathological alterations in *Cirrhinus mrigala* [64]. The significantly lower values of body mass of fish exposed to higher salinity levels at days 60 and 90 could be related to induction of oxidative stress leading to disruption of physiological homeostasis. Previously, significant growth retardation due to different salinity levels in *C. catla* (from 1 ppt onwards), *L. rohita* (from 3 ppt onwards) and *C. mrigala* (from 4 ppt onwards) has been recorded by Hoque et al. [50].

A significant decline in key hematological biomarkers- i-e, erythrocytic count, lymphocyte count, hemoglobin concentration, and hematocrit—was observed in mrigal carp reared in brackish water conditions. These findings are consistent with previous studies suggesting that hematological parameters serve as sensitive and reliable indicators of physiological stress in both aquatic as well as terrestrial organisms [65,66]. Alterations in these blood indices often reflect impaired oxygen transport capacity and immune function, commonly associated with environmental stressors such as salinity fluctuations [67,68]. The observed decline in hematological parameters in this study may be attributed to stress-induced suppression of hematopoietic activity, increased erythrocyte lysis, and oxidative damage to hemoglobin under elevated salinity conditions [64,69–71]. In this study, the elevated leukocyte and neutrophil counts observed in mrigal carp kept in brackish water may reflect the activation of innate immune responses and the presence of inflammatory or injurious stimuli affecting various visceral organs. These hematological changes are consistent with findings from earlier studies, which reported increased leukocyte and neutrophil levels—alongside reductions in erythrocyte count, lymphocyte count, hematocrit, and hemoglobin—as typical physiological responses to oxidative stress triggered by aquatic pollutants or environmental contaminants [11,64,72,73]. Previous studies have similarly documented reductions in hematological indicators—erythrocyte count, hemoglobin, hematocrit, and lymphocyte levels—in various fish species exposed to environmental stressors such as rohu (*Labeo rohita*) [74], *Catla catla* (Theila) [75] and *Gabelion catla* [76]. Moreover, earlier studies have reported elevated white blood cell (WBC) counts in butter catfish (*Ompok bimaculatus*), Nile tilapia [77], and *Cyprinus carpio* [78] exposed to increased salinity levels, indicating an enhanced immune response under salinity-induced stress [79]. The hematological abnormalities observed in this study may also be attributed to inflammation within the vascular system, leading to excessive generation of free radicals, red blood cell destruction, and impaired function of hematopoietic tissues [65,80]. Additionally, disruption of osmoregulatory processes in the gills under high salinity conditions may have resulted in inadequate oxygen delivery to blood-forming organs in mrigal carp. These physiological disruptions collectively contribute to hematological imbalances. Conversely, several previous studies have reported elevated hematological values in certain fish species such as *Cyprinus carpio* [78] and *Notopterus notopterus* [81], suggesting that responses to salinity stress can vary depending on species-specific adaptability and tolerance thresholds. The elevated neutrophil percentage and white blood cell (WBC) counts observed in mrigal carp may also be associated with stress conditions induced by exposure to brackish water with elevated salinity levels.

The quantity of different serum enzymes such as ALP, AST and ALT increased considerably in in mrigal carp reared at high salinity levels. Significantly lower quantity of serum albumin, glucose and triglycerides was measured in our study in mrigal carp reared in brackish water at high salinity levels. In our study, the quantity of serum cholesterol and LDH increased significantly. Monitoring of various serum biochemistry parameters are considered as reliable, precise and exact biomarkers to know the pathophysiological status of several tissues in aquatic and terrestrial organisms exposed to several toxicants and abrupt changes in ecological climatic conditions [47,82,83]. The elevated level of cholesterol and LDH along with lower values of glucose in mrigal carp might be related to oxidative stress induced by brackish water [70]. Creatinine, a key byproduct of purine metabolism, is widely recognized as an important biomarker for evaluating renal and muscular function, particularly under conditions of oxidative stress [84]. In the present study, the significantly elevated levels of urea and creatinine in mrigal carp exposed to brackish water suggest impaired kidney function, likely resulting from salinity-induced renal damage. In earlier studies, it has also been reported that the quantity of ALP, AST and ALT increased significantly with increasing trend of salinity in *Cyprinus carpio* [85], and *Labeo rohita* [86]. Previously, increased amount of cholesterol, triglycerides and LDH due to higher salinity level in different fish species reared in brackish water like *Notopterus notopterus* has also recorded [81,85,86]. Furthermore, increased quantity of glucose different species of fish like *Cyprinus carpio* [87], *Colossoma macropomum* [88], *Labeo rohita* [86] and *Clarias gariepinus* [89]. Increased quantity of urea and creatinine in various fish species including *Notopterus notopterus* [81] and *Clarias gariepinus* [89] due to various salinity levels has also been reported. Similar results regarding the quantity of albumin protein have been reported in different fish species like *Cyprinus carpio* [87] and *Labeo rohita* [86] related to increased salinity values.

In the current study, elevated levels of TBARS were observed in the erythrocytes of mrigal carp exposed to increased salinity, indicating enhanced lipid peroxidation. Concurrently, the concentrations of key antioxidant enzymes—namely glutathione (GSH), superoxide dismutase (SOD), and catalase (CAT)—declined markedly, reflecting a compromised antioxidant defense system under salinity-induced oxidative stress. To date, no published data exist on the status of TBARS and antioxidant enzymes specifically in the erythrocytes of *Cirrhinus mrigala* reared in brackish water across different salinity regimes. This study, therefore, represents the first report to evaluate oxidative stress and antioxidant enzyme activity in isolated erythrocytes of mrigal carp under such conditions. The observed increase in TBARS may be due to over production of reactive oxygen species triggered by elevated salinity, overwhelming the cellular antioxidant capacity [70,90,91]. It is well established that the overproduction of reactive oxygen species (ROS) initiates lipid peroxidation, resulting in structural damage to cellular membranes and the subsequent formation of thiobarbituric acid reactive substances (TBARS) in animal tissues [92,93]. Previously, higher ROS and TBARS levels in *Dicentrarchus labrax* [94], *Catla catla* [95] and Esturine fish [96] have been recorded due to higher salinity levels. The elevated oxidative stress markers, along with reduced levels of antioxidant enzymes in the erythrocytes of fish reared in brackish water with varying salinity levels, may be attributed to damage induced by reactive oxygen species (ROS). These free radicals likely caused cellular injury to red blood cells and contributed to the depletion of the antioxidant defense system [97–100]. Previously, lower values of SOD and CAT in fish (*Cyprinus carpio)* have been reported [101]. Increased oxidative stress along with decreased antioxidant enzymes are suggestive of decreased potential of status of defense mechanisms of fresh water fish (mrigal carp) reared in brackish water [51,102,103].

In previous studies, various histopathological abnormalities have been observed in gills including epithelial necrosis, telangiectasia at the tips of secondary lamellae, lifting of lamellar epithelium, hypertrophy in chloride cells along with fusion of secondary lamellae in fish species namely *Pangasionodon hypophthalamus* [104], *Lates reticulatus* [105], *Catla catla* [106], *Rutilus rutilus* [107] and *Scatophagus argus* [108]. Histopathological examination in the present study revealed notable alterations in the brain and heart tissues of mrigal carp reared in brackish water, including neuronal atrophy, neuronal necrosis, and microgliosis in the brain, as well as degeneration and disorganization of cardiac muscle fibers. The histopathological alterations in multiple visceral organs of fish reared in saline water might be due to induction of oxidative stress [100,109,110]. To date, no published reports are available detailing the histopathological effects of brackish water on the heart and brain of *Cirrhinus mrigala*, making this study a novel contribution in this area. These alterations may be attributed to elevated salinity levels and associated disruption of osmoregulatory mechanisms in the exposed fish. Furthermore, similar microscopic lesions—such as the presence of melano-macrophage centers, renal tubular degeneration, and necrotic cells—have been reported in *Pangasianodon hypophthalmus* under salinity-induced stress [104], supporting the notion that excessive salinity can induce widespread pathological damage in vital organs. Previous studies have reported a range of histopathological abnormalities in the liver of various fish species, including *Pangasianodon hypophthalmus*, such as hepatic congestion, fatty degeneration, hepatocellular necrosis, and nuclear hypertrophy [104] and *Channa punctatus* [111].

## Conclusion

The results on integrated assessment of salinity induced deleterious effects in terms of analysis of hemato-biochemistry, oxidative stress biomarkers, histopathology and erythrocytic toxicity in Mrigal carp indicated that elevated salinity in fresh water can provoke oxidative stress, impair haematological and biochemical homeostasis, suppress antioxidant defense mechanisms in erythrocytes, and induce microscopic ailments in different visceral organs of fresh water fish in a time dependent response. Moreover, hematological and biochemical indices are useful and reliable tools and provide extensive information about the early screening of variety of toxicants.

## Supporting information

S1 File Effects of Brackish water on growth, blood-biochemistry and oxidative stress of Mrigal carp. (XLSX)
